# Evaluating the role of astragalus polysaccharide in modulating immune infiltration and enhancing prognostic biomarkers in pediatric acute myeloid leukemia

**DOI:** 10.3389/fphar.2025.1538888

**Published:** 2025-04-07

**Authors:** Min-Hui He, Xian-Hui Zhang, Ji-Hong Zhang, Jie Pan, Chao Yang

**Affiliations:** ^1^ Clinical laboratory center, Children’s Hospital of Shanxi Province (Women Health Center of Shanxi Province), Taiyuan, China; ^2^ Department of Internal Medicine, Children’s Hospital of Shanxi Province (Women Health Center of Shanxi Province), Taiyuan, China; ^3^ Hematology Research Laboratory, Shengjing Hospital Affiliated to China Medical University, Shenyang, China; ^4^ Department of Pathology, Stanford University School of Medicine, Palo Alto, CA, United States; ^5^ Department of Clinical Laboratory, Taiyuan Central Hospital (Peking University First Hospital Taiyuan Hospital), Taiyuan, Shanx, China

**Keywords:** APS, pediatric acute myeloid leukemia (PAML), GEO database, PPI network, GO/KEGG, immune infiltration analysis, prognostic biomarker

## Abstract

**Background:**

Childhood acute myeloid leukemia (AML) constitutes a significant proportion of pediatric malignancies, with current treatment options remaining limited. This study aimed to investigate the role of Astragalus polysaccharide (APS) in immune infiltration and prognosis of pediatric AML.

**Methods:**

Differentially expressed genes (DEGs) were identified from the GEO database (dataset GSE2191), and APS-related genes (APSRGs) were obtained from the Swiss Target Prediction platform. DEGs with |logFC| > 1 and p < 0.05 were intersected with APSRGs to identify APS-related differentially expressed genes (APSRDEGs), visualized using a Venn diagram. A protein-protein interaction (PPI) network analysis was conducted to identify hub genes. Gene Ontology (GO) and KEGG enrichment analyses were performed to determine biological processes (BP), cellular components (CC), molecular functions (MF), and relevant pathways associated with the hub genes. Correlation analysis, receiver operating characteristic (ROC) curve analysis, and immune infiltration analysis were conducted to assess the relationship between hub genes and pediatric AML.

**Results:**

The GSE2191 dataset was divided into pediatric AML (PAML) and control groups. A total of 1,881 DEGs were identified, of which 20 were APSRDEGs. PPI network analysis revealed that 13 APSRDEGs were interconnected, and nine hub genes were identified: *CASP3*, *PTPRC*, *ELANE*, *HMOX1*, *CHUK*, *FLT1*, *JAK3*, *CTSL*, and *AURKA*. GO and KEGG enrichment analyses indicated that these genes were significantly associated with key biological processes, cellular components, molecular functions, and pathways involved in AML. ROC curve analysis revealed that the expression levels of the nine hub genes differed significantly between the PAML and control groups. Immune infiltration analysis demonstrated a strong correlation between several hub genes and immune cells, with HMOX1 showing the strongest positive correlation with neutrophils.

**Conclusion:**

This study identified nine hub genes related to APS in pediatric AML. These findings suggest that APS may significantly affect immune infiltration and prognosis in pediatric AML, highlighting its potential as a therapeutic modulator for the disease.

## 1 Introduction

Acute myeloid leukemia (AML) is a malignant hematological disorder that primarily affects myeloid stem cells, leading to the uncontrolled proliferation of immature blood cells. It represents one of the most common forms of leukemia in adults and children, with pediatric acute myeloid leukemia (PAML) accounting for approximately 20% of childhood leukemia cases (Hiroto et al., 2019). Despite significant advances in treatment strategies, including intensive chemotherapy and hematopoietic stem cell transplantation, the prognosis for pediatric AML remains poor. One of the most significant challenges in managing AML is the high relapse rate, with approximately one-third to one-half of patients experiencing recurrence after initial remission (Stevens et al., 2017). This relapse is often due to the persistence of leukemic stem cells that evade conventional therapies. Consequently, there is an urgent need for alternative or adjunctive therapies that could improve the prognosis of PAML patients. Astragalus polysaccharide (APS), a bioactive compound derived from the traditional Chinese herb Astragalus membranaceus, has demonstrated various immune-modulating and anti-tumor properties. This study aims to investigate the potential of APS in modulating immune infiltration and enhancing prognostic biomarkers in pediatric AML, offering new insights into the possibility of combining APS with existing therapies to reduce relapse rates and improve overall outcomes.

Astragalus polysaccharide (APS), a principal bioactive component derived from the traditional Chinese medicinal herb Astragalus membranaceus, has demonstrated a wide range of pharmacological activities, including potent antitumor effects. While the anti-cancer properties of APS have been extensively studied in various malignancies, such as colon, liver, lung, and gastric cancers, its potential role in leukemia, particularly AML, remains less explored. Recent studies have suggested that APS exerts significant inhibitory effects on cancer cell proliferation, migration, and invasion, primarily by modulating key signaling pathways involved in tumor growth, apoptosis, and immune responses. For example, APS has been shown to induce apoptosis in various cancer cell lines, including those from colon and breast cancers, by activating intrinsic apoptotic pathways and downregulating anti-apoptotic proteins (Di et al., 2009).

In the context of leukemia, APS has demonstrated promising potential in regulating the immune microenvironment and enhancing the immune response against tumor cells. Studies indicate that APS may improve the efficacy of chemotherapy by enhancing immune cell activity, particularly by stimulating the proliferation and activation of natural killer (NK) cells and T lymphocytes. These immune cells play a crucial role in the elimination of leukemic cells, and APS has been shown to augment their cytotoxic functions. Additionally, APS has been observed to reduce the inflammatory responses typically associated with leukemia, potentially mitigating the adverse effects of chemotherapy and improving the overall therapeutic response.

While conventional treatments for AML, including chemotherapy and hematopoietic stem cell transplantation, remain the standard of care, these therapies are often limited by issues such as drug resistance, toxicity, and high relapse rates. APS, with its ability to modulate immune responses and enhance the sensitivity of leukemic cells to chemotherapy, presents a promising alternative or adjunct to traditional treatments. By targeting multiple pathways, including apoptosis, immune modulation, and inflammation, APS offers a multi-targeted approach that could help overcome the limitations of current therapies. This makes APS a potential candidate for combination therapies, particularly in addressing the challenge of relapse in pediatric AML. However, despite its potential, the precise molecular mechanisms underlying the anticancer effects of APS in leukemia, especially AML, require further investigation.

Network pharmacology provides a systemic approach to exploring complex interactions between multiple components and targets, offering valuable insights into disease mechanisms and therapeutic strategies (Hopkins, 2008). Tumorigenesis is driven by multi-gene interactions, necessitating multi-target therapeutic approaches. Network pharmacology facilitates the identification of novel therapeutic targets through integrative analyses, relying on extensive data to uncover potential mechanisms of action. Network pharmacology is particularly relevant given the multi-gene regulatory nature of leukemia and the corresponding need for multi-target treatment strategies. However, the application of network pharmacology to investigate the effects of APS on AML remains underexplored. To elucidate the comprehensive mechanisms of APS in childhood AML, we employed a network pharmacology approach to identify potential targets and functional pathways. This study integrated pediatric AML datasets from the Gene Expression Omnibus (GEO) with APS-related genes (APSRGs) identified using the SwissTargetPrediction platform. Differentially expressed genes (DEGs) were analyzed to identify APS-related differentially expressed genes (APSRDEGs). Hub genes were identified using a protein-protein interaction (PPI) network, followed by Gene Ontology (GO) and KEGG pathway enrichment analyses. Further, ROC curve analyses and immune infiltration studies were performed to assess these genes diagnostic and prognostic significance. Ultimately, we identified key diagnostic and immune infiltration-associated genes for childhood AML.

## 2 Materials and methods

### 2.1 Data download

Data for PAML were downloaded from the GEO database (http://www.ncbi.nlm.nih.gov/geo/) (Davis and Meltzer, 2007). The dataset GSE2191 (Yagi et al., 2003) comprises samples derived from *Homo sapiens*, with tissue sources including bone marrow and peripheral blood. The platform used for GSE2191 was GPL8300, with detailed information provided in Table 1. This dataset includes 54 PAML and 4 control samples, all incorporated into this study. The dataset was standardized using the R package limma (Ritchie et al., 2015). Probe annotations were normalized and standardized, and boxplots depicting data before and after standardization were generated.

**TABLE 1:** GEO microarray chip information.

	GSE2191
Platform	GPL8300
Type	Array
Species	*Homo sapiens*
Tissue	Bone marrow or peripheral blood
Samples in the PAML group	54
Samples in the Control group	4
Reference	PMID: 12738660

GEO, gene expression omnibus; PAML, pediatric acute myeloid leukemia.

### 2.2 Astragalus polysaccharide target prediction

The PubChem database (https://pubchem.ncbi.nlm.nih.gov/) (Kim et al., 2021), a comprehensive resource for chemical information, was utilized to retrieve the Simplified Molecular Input Line Entry System (SMILES) expression of APS. Target prediction for APSRGs was performed using the SwissTargetPrediction online tool (http://swisstargetprediction.ch/) (Daina et al., 2019) based on compound structure. The specific details are summarized in Supplementary Table S1. Subsequently, an APS-target network was constructed to visualize interactions between APS and its predicted targets.

### 2.3 Identification of APS-related differentially expressed genes in pediatric AML

Samples in dataset GSE2191 were divided into two groups: the PAML group and the control group. Differential gene expression analysis was conducted using the R package limma. Genes were identified as differentially expressed if they met the criteria of |logFC| > 1 and p < 0.05. Upregulated genes were defined as those with logFC > 1 and p < 0.05, while downregulated genes were those with logFC < −1 and p < 0.05. The Benjamini–Hochberg (BH) method was used for p-value adjustment.

To identify APSRDEGs associated with pediatric AML, DEGs from dataset GSE2191 were intersected with APSRGs. Only overlapping genes meeting the DEG criteria were considered APSRDEGs.

### 2.4 PPI network construction and hub gene identification

The STRING database (http://string-db.org/) (Szklarczyk et al., 2019) was employed to construct a PPI network for APSRDEGs, with a minimum interaction confidence score of 0.400 (medium confidence). Local regions of the PPI network, representing potential molecular complexes with specific biological functions, were identified for further analysis. Hub genes were selected using the CytoHubba plugin in Cytoscape (Shannon et al., 2003; Chin et al., 2014), applying five algorithms: Maximal Clique Centrality (MCC), Maximum Neighborhood Component (MNC), Degree, Edge Percolated Component (EPC), and Closeness (Yang et al., 2019). Top-ranking APSRDEGs identified by all five algorithms were intersected, and a Venn diagram was generated to determine the Hub genes.

### 2.5 GO and KEGG pathway enrichment analysis

GOanalysis (Mi et al., 2019) was performed to investigate the biological processes (BP), cellular components (CC), and molecular functions (MF) associated with Hub genes. Kyoto Encyclopedia of Genes and Genomes (KEGG) pathway analysis (Kanehisa and Goto, 2000), was conducted to explore the involvement of Hub genes in disease pathways. The R package clusterProfiler (Yu et al., 2012) was used for both GO and KEGG enrichment analyses, with statistical significance defined as asjusted p-value < 0.05 and FDR (q-value) < 0.25. The Benjamini–Hochberg (BH) method was used for p-value correction.

### 2.6 Differential expression validation and ROC curve analysis of hub genes

Group comparison plots were generated to validate the differential expression of Hub genes between the PAML and control groups. The diagnostic performance of Hub genes was evaluated using ROC curves, plotted with the R package pROC. The area under the curve (AUC) was calculated to quantify diagnostic accuracy. AUC values > 0.9 indicated high accuracy, values between 0.7 and 0.9 indicated moderate accuracy, and values between 0.5 and 0.7 indicated low accuracy.

### 2.7 Hub gene correlation and functional similarity analysis

The Spearman algorithm was used to assess correlations between the expression levels of Hub genes in dataset GSE2191. Genes with the strongest correlations were identified using the R package ggplot2. Correlation strength was categorized as follows: weak or no correlation (|r| < 0.3), weak correlation (0.3 ≤ |r| < 0.5), moderate correlation (0.5 ≤ |r| < 0.8), and strong correlation (|r| ≥ 0.8).

Functional similarities among Hub genes were analyzed using GOSemSim (Yu et al., 2010), which calculates functional similarity scores based on GO annotations.

### 2.8 Immune infiltration analysis (ssGSEA)

The relative abundance of immune cell infiltration in each sample was assessed using the single-sample gene-set enrichment analysis (ssGSEA) (Xiao et al., 2020) method. Immune cell infiltration matrices were generated for dataset GSE2191, and correlations between Hub genes and immune cell infiltration levels were analyzed using the Spearman algorithm.

### 2.9 Statistical analysis

All statistical analyses were performed using R software (version 4.3.0). For comparisons of continuous variables between two groups, normally distributed variables were analyzed by independent Student’s T-Test. In contrast, non-normally distributed variables were analyzed using the Mann-Whitney U test (Wilcoxon rank-sum test). For comparisons involving three or more groups, the Kruskal–Wallis test was applied. Spearman correlation coefficients were calculated for association analyses. Statistical significance was set at a two-sided p < 0.05 unless otherwise specified.

## 3 Results

### 3.1 Technology roadmap

**FIGURE 1:**
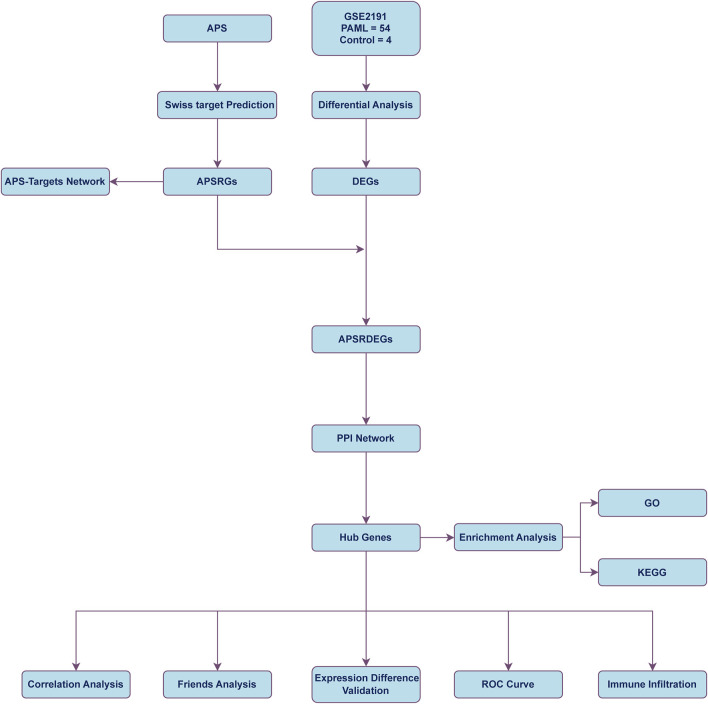
Flow chart for the comprehensive analysis of APS PAML, pediatric acute myeloid leukemia; DEGs, differentially expressed genes; APS, astragalus polysacharin; APSRGs, astragalus polysacharin-related genes. APSRDEGs, astragalus polysacharin-related differentially expressed genes; ROC, receiver operating characteristic; GO, gene ontology; KEGG, kyoto encyclopedia of genes and genomes; PPI, protein-protein interaction.

Figure 1 illustrates the comprehensive workflow of the study, outlining the key stages of the data analysis process and the methodologies employed to explore the role of Astragalus polysaccharide (APS) in pediatric acute myeloid leukemia (PAML). The roadmap begins with the identification of differentially expressed genes (DEGs) from the GSE2191 dataset, followed by the integration of APS-related genes (APSRGs) identified through the Swiss Target Prediction platform. The intersecting DEGs and APSRGs were used to identify APS-related differentially expressed genes (APSRDEGs), which were then analyzed through protein-protein interaction (PPI) network analysis to identify hub genes.

Subsequently, Gene Ontology (GO) and Kyoto Encyclopedia of Genes and Genomes (KEGG) pathway enrichment analyses were performed to explore the biological processes, molecular functions, and relevant pathways associated with the identified hub genes. The inclusion of receiver operating characteristic (ROC) curve analysis and immune infiltration studies further helped assess the diagnostic and prognostic significance of these hub genes in the context of PAML.

Figure 1 also highlights the sequential integration of these analyses, from data extraction and gene identification to in-depth statistical and immune-related assessments, emphasizing the methodological framework that underpins the study’s aim to identify key biomarkers and therapeutic targets for APS in PAML. This roadmap serves as a visual guide to understanding how the various data sets and analytical tools converge to uncover the complex interactions between APS and the immune microenvironment in AML. The visual representation is critical for understanding the logical flow of the study, from gene identification to pathway analysis, immune infiltration, and the final determination of potential therapeutic applications of APS in AML.

### 3.2 Target prediction of Astragalus Polysaccharides

**FIGURE 2:**
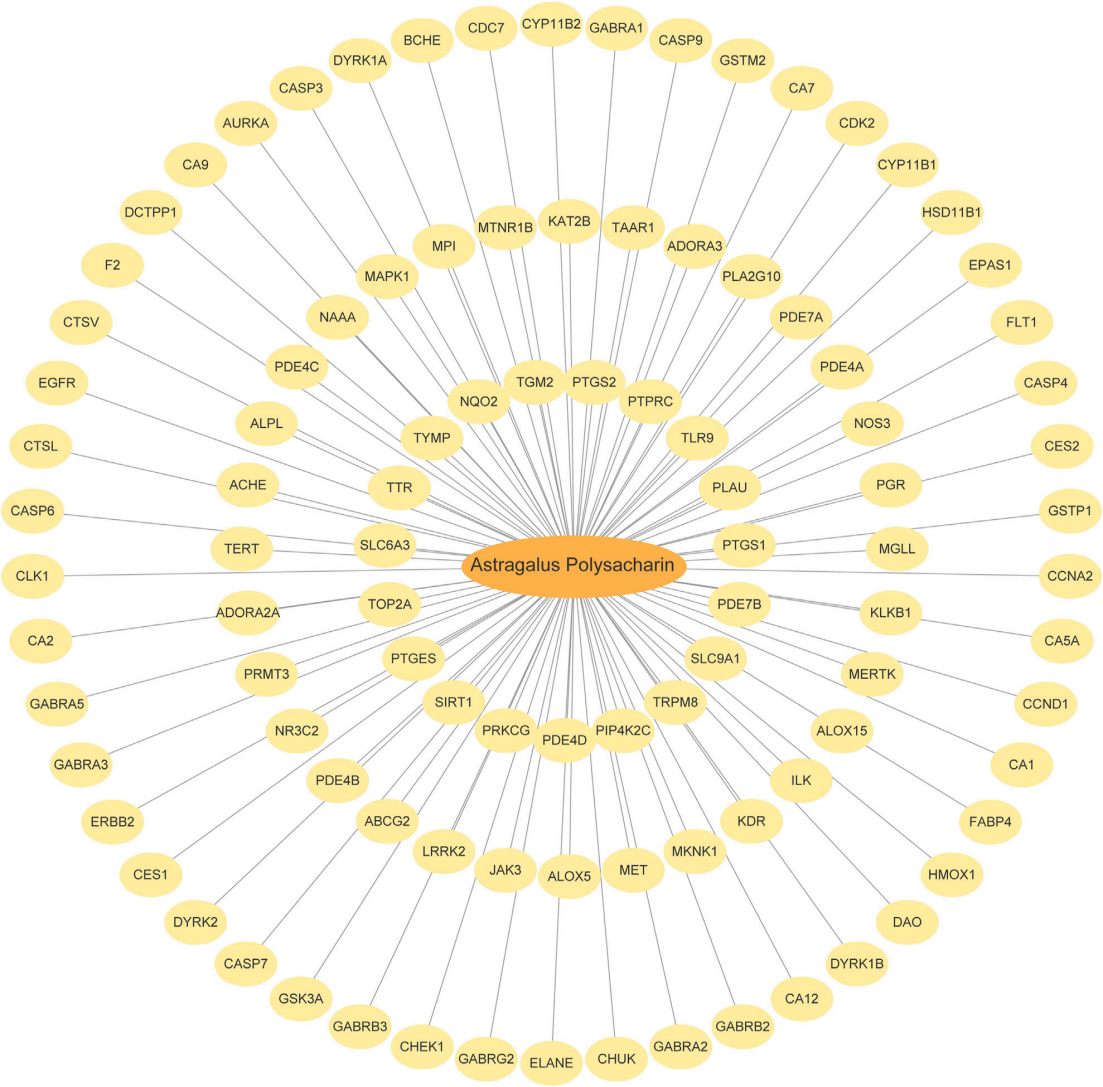
APS and Targets Interaction Network. The orange oval is Astragalus polysaccharide, and the yellow oval is SwissTargetPrediction target.

The English term Astragalus Polysaccharide (APS) was used as the keyword to search in the PubChem database. APS is a complex polysaccharide composed of multiple monosaccharide units connected by glycosidic bonds, so it is not possible to fully describe its structure with a simple chemical formula. The specific chemical formula may vary slightly depending on the different extracts, for example, shorter APS molecules are roughly between C_18_H_32_O_16_ to C_24_H_42_O_21_. Target prediction was conducted using the Swiss Target Prediction platform, identifying 101 APS-related genes (APSRGs). These genes were visualized using a Cytoscape network diagram (Figure 2), with detailed information provided in Supplementary Table S2.

### 3.3 Standardization of the pediatric acute myeloid leukemia dataset

**FIGURE 3:**
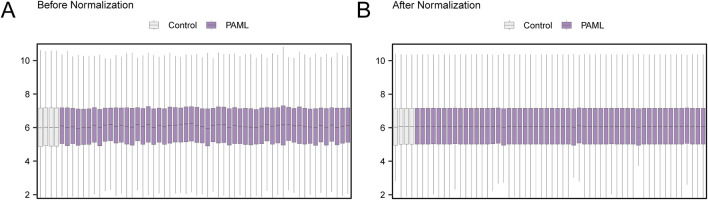
Normalization of GSE2191. **(A)** Boxplot of GSE2191 distribution in the dataset before normalization. **(B)** Boxplot of GSE2191 distribution of the standardized dataset. Control group (gray) and childhood acute myeloid leukemia (PAML) group (purple).

The dataset GSE2191 was standardized using the R package limma. Boxplots were generated comparing the expression distributions before and after standardization (Figures 3A, B).

### 3.4 Identification of APS-related differentially expressed genes in pediatric acute myeloid leukemia

**FIGURE 4:**
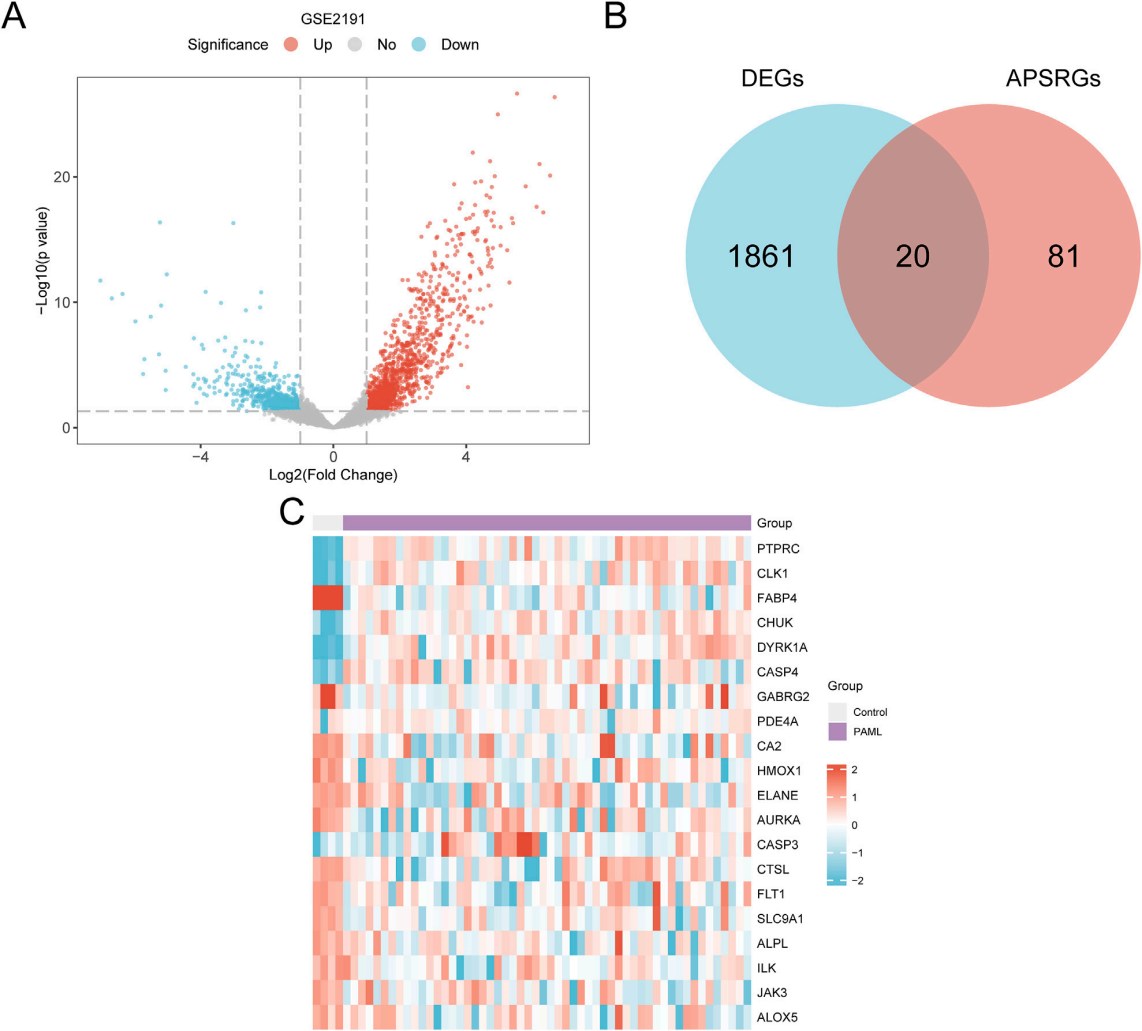
Differential Gene Expression Analysis. **(A)** Volcano plot of differentially expressed genes analysis between PAML and Control groups in dataset GSE2191. **(B)** DEGs in dataset GSE2191, APSRGs Venn diagram. **(C)** Heat map of APSRDEGs in dataset GSE2191. The PAML group is in purple, and the Control group is in gray. In the heat map, red represents high expression, blue represents low expression, and the depth of color represents the degree of expression.

The data of dataset GSE2191 were divided into the PAML and control groups. A total of 1,881 differentially expressed genes (DEGs) meeting the thresholds |logFC| > 1 and p < 0.05 were identified. Of these, 1,119 genes were upregulated, and 762 were downregulated. A volcano plot summarizing these results was generated (Figure 4A).

The intersection of APSRGs and DEGs was visualized using a Venn diagram and yielded 20 APS-related differentially expressed genes (APSRDEGs): *ALOX5*, *ALPL*, *FLT1*, *GABRG2*, *HMOX1*, *ILK*, *JAK3*, *PDE4A*, *PTPRC*, and *SLC9A1* (Figure 4B). Heatmap analysis of APSRDEG expression differences between the groups was performed using the heatmap package (Figure 4C).

### 3.5 Construction of a PPI network and hub gene screening

**FIGURE 5:**
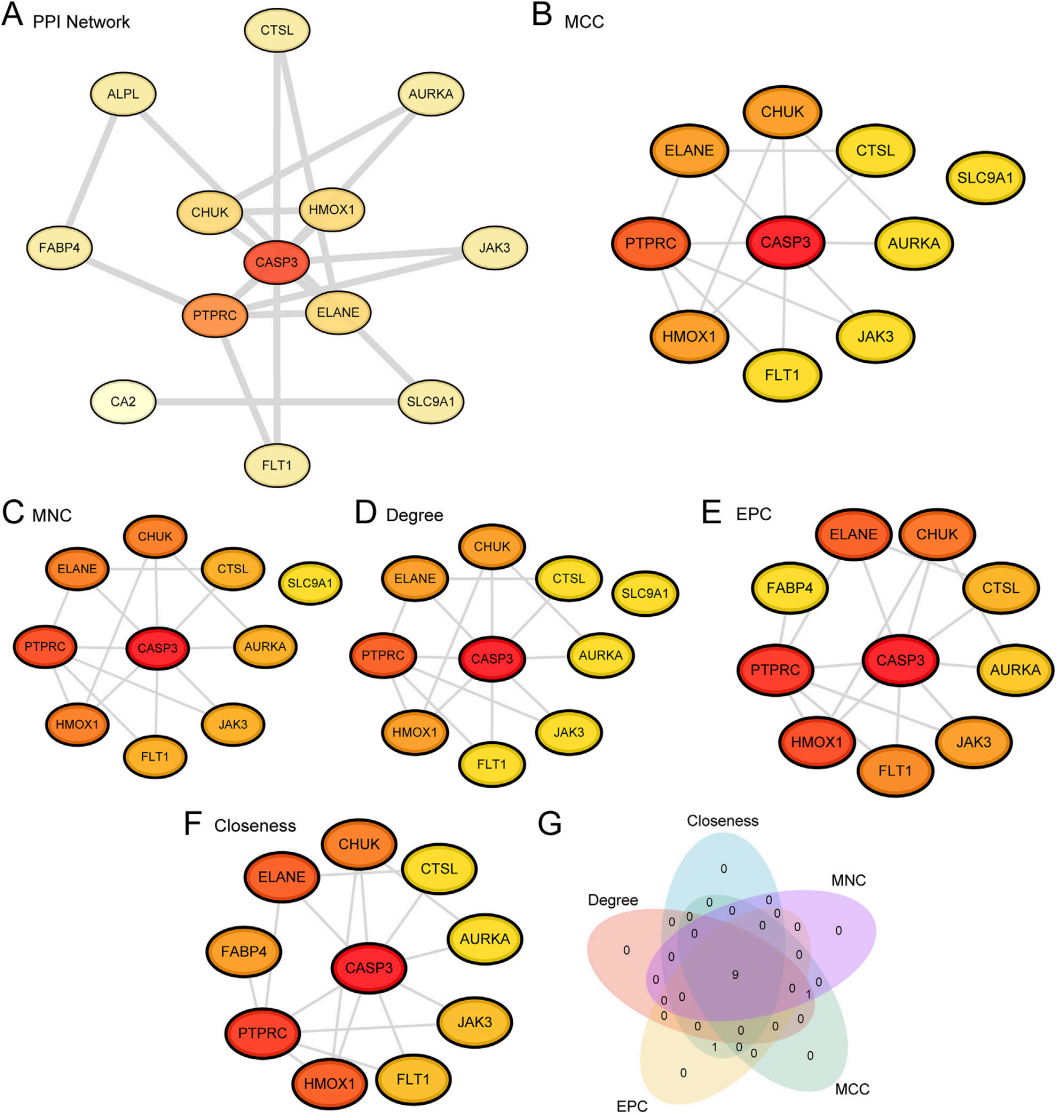
PPI Network and Hub Genes Analysis. **(A)** PPI Network of APSRDEGs calculated from the STRING database. Only 13 of the 20 APSRDEGs formed significant interactions with other proteins, highlighting the most interconnected genes in the network. **(B–F)** PPI Network of TOP10 APSRDEGs calculated by five algorithms of CytoHubba plug-in, including MCC **(B)**, MNC **(C)**, Degree **(D)**, EPC **(E)** and Closeness **(F)**. **(G)**. APSRDEGs Venn diagram of TOP10 for the five algorithms of the CytoHubba plugin.

Protein-protein interaction (PPI) analysis for the 20 APSRDEGs was conducted using the STRING database. However, as depicted in Figure 5A, only 13 of the 20 APSRDEGs formed significant interactions with other proteins, highlighting the most interconnected genes in the network. They are *ALPL*, *AURKA*, *CA2*, *CASP3*, *CHUK*, *CTSL*, *ELANE*, *FABP4*, *FLT1*, *HMOX1*, *JAK3*, *PTPRC*, and *SLC9A1*. The remaining seven APSRDEGs, while identified in the initial analysis, did not show strong or significant interactions in the PPI network and were not included in Figure 5A. This subset of genes still holds potential for further investigation, but their lower interaction scores in the network suggest that their roles may be more isolated or indirect. Scores for these genes were calculated using five CytoHubba algorithms (MCC, MNC, Degree, EPC, and Closeness), and the top 10 ranked genes for each algorithm were visualized as PPI networks (Figures 5B–F). A Venn diagram identified nine hub genes shared across the algorithms: *CASP3*, *PTPRC*, *ELANE*, *HMOX1*, *CHUK*, *FLT1*, *JAK3*, *CTSL*, and *AURKA* (Figure 5G).

### 3.6 Gene ontology (GO) and pathway (KEGG) enrichment analysis

**FIGURE 6:**
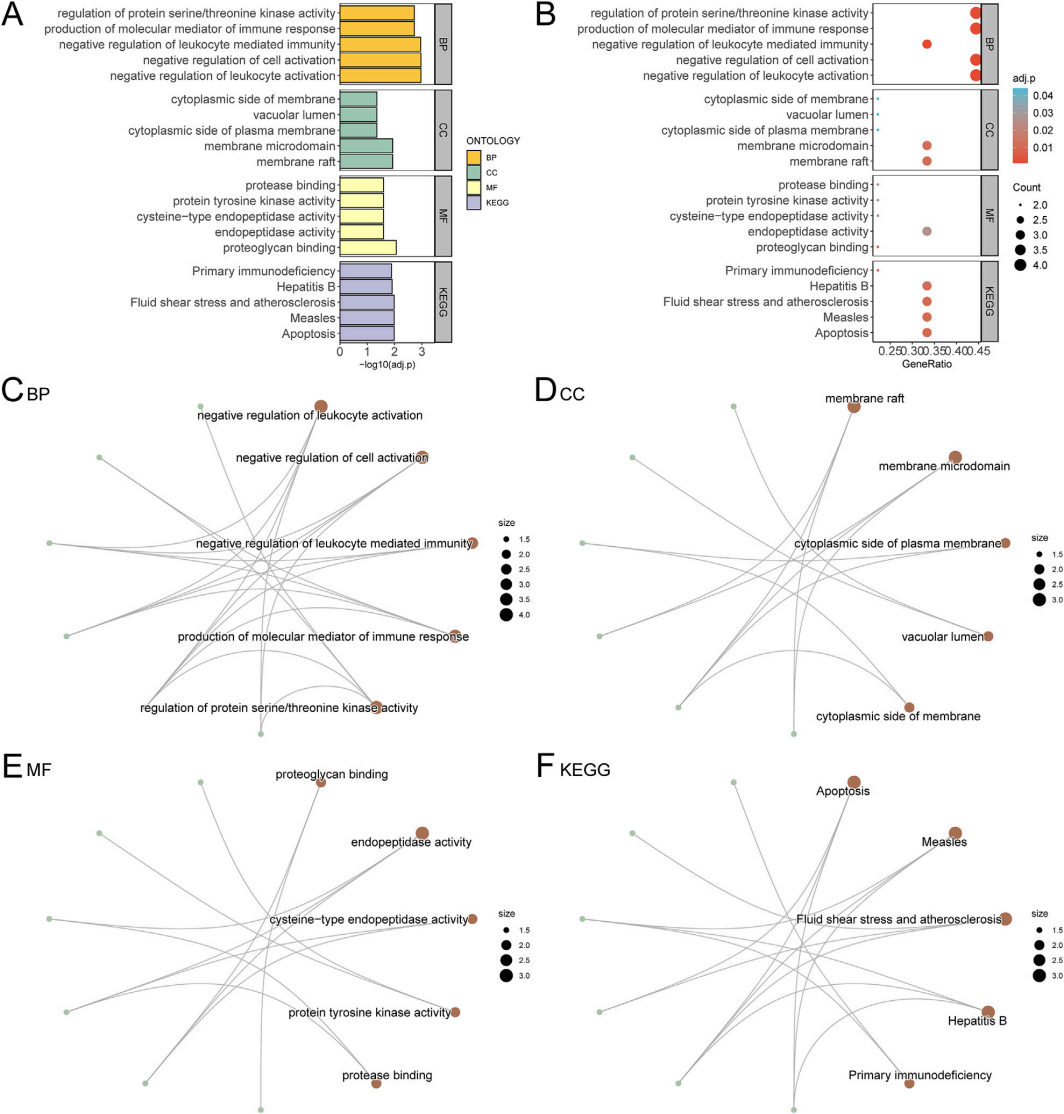
GO and KEGG Enrichment Analysis for Hub Genes. **(A)** The results of GO and KEGG of Hub Genes are shown in bar chart **(A)** and bubble chart **(B)** Biological process (BP), cell component (CC), molecular function (MF) and biological pathway (KEGG), GO terms and KEGG terms are on the ordinate. **(C–F)** GO and KEGG results of Hub Genes network diagram: BP **(C)**, CC **(D)**, MF **(E)** and KEGG **(F)**. The brown nodes represent entries, the green nodes represent molecules, and the lines represent the relationship between entries and molecules. The bubble size in the bubble plot represents the number of genes, and the color of the bubble represents the size of the adj. p value, the reder the color, the smaller the adj. p value, and the bluer the color, the larger the adj. p value. The screening criteria for GO and KEGG were adj. p value < 0.05 and FDR value (q value) < 0.25, and the p value correction method was Benjamini–Hochberg (BH).

Gene Ontology (GO) and KEGG pathway enrichment analyses were performed for the nine hub genes, with results summarized in Table 2. Key biological processes (BP) included negative regulation of leukocyte activation and immune-mediated processes. Cellular components (CC) such as membrane rafts and vacuolar lumen were enriched, along with molecular functions (MF) like proteoglycan binding and endopeptidase activity. Enriched KEGG pathways included apoptosis, hepatitis B, and primary immunodeficiency. Results were visualized using bar and bubble plots (Figures 6A, B) and network diagrams (Figures 6C–F).

**TABLE 2:** Result of GO and KEGG enrichment analysis for hub genes.

Ontology	ID	Description	GeneRatio	BgRatio	p-value	p.adjust	q-value
BP	GO:0002695	Negative regulation of leukocyte activation	4/9	202/18614	1.63E-06	1.07E-03	3.63E-04
BP	GO:0050866	Negative regulation of cell activation	4/9	225/18614	2.50E-06	1.07E-03	3.63E-04
BP	GO:0002704	Negative regulation of leukocyte mediated immunity	3/9	68/18614	3.86E-06	1.10E-03	3.73E-04
BP	GO:0002440	Production of molecular mediator of immune response	4/9	328/18614	1.11E-05	1.89E-03	6.38E-04
BP	GO:0071900	Regulation of protein serine/threonine kinase activity	4/9	369/18614	1.77E-05	1.89E-03	6.38E-04
CC	GO:0045121	Membrane raft	3/9	323/19518	3.50E-04	1.17E-02	6.88E-03
CC	GO:0098857	Membrane microdomain	3/9	324/19518	3.53E-04	1.17E-02	6.88E-03
CC	GO:0009898	Cytoplasmic side of plasma membrane	2/9	159/19518	2.29E-03	4.41E-02	2.60E-02
CC	GO:0005775	Vacuolar lumen	2/9	176/19518	2.79E-03	4.41E-02	2.60E-02
CC	GO:0098562	Cytoplasmic side of membrane	2/9	193/19518	3.35E-03	4.41E-02	2.60E-02
MF	GO:0043394	Proteoglycan binding	2/9	36/18369	1.33E-04	8.66E-03	2.67E-03
MF	GO:0004175	Endopeptidase activity	3/9	428/18369	9.50E-04	2.53E-02	7.79E-03
MF	GO:0004197	Cysteine-type endopeptidase activity	2/9	118/18369	1.43E-03	2.53E-02	7.79E-03
MF	GO:0004713	Protein tyrosine kinase activity	2/9	137/18369	1.92E-03	2.53E-02	7.79E-03
MF	GO:0002020	Protease binding	2/9	138/18369	1.95E-03	2.53E-02	7.79E-03
KEGG	hsa04210	Apoptosis	3/9	136/8662	2.97E-04	1.03E-02	6.33E-03
KEGG	hsa05162	Measles	3/9	138/8662	3.10E-04	1.03E-02	6.33E-03
KEGG	hsa05418	Fluid shear stress and atherosclerosis	3/9	139/8662	3.16E-04	1.03E-02	6.33E-03
KEGG	hsa05161	Hepatitis B	3/9	162/8662	4.97E-04	1.22E-02	7.45E-03
KEGG	hsa05340	Primary immunodeficiency	2/9	38/8662	6.62E-04	1.30E-02	7.94E-03

GO, gene ontology; BP, Biological Process; CC, Cellular Component; MF, molecular function; KEGG, kyoto encyclopedia of genes and genomes.

#### 3.6.1 Biological processes (BP)

The GO analysis revealed that the hub genes were significantly involved in several important biological processes relevant to AML, such as the negative regulation of leukocyte activation and immune-mediated processes. These processes are critical for understanding how APS may enhance immune surveillance in the AML microenvironment, potentially aiding in the elimination of leukemic cells. The activation and modulation of immune responses are key to improving the body’s ability to target and control leukemia. Additionally, regulation of protein serine/threonine kinase activity was identified, which is involved in several crucial signaling pathways that regulate cell growth and apoptosis in AML.

#### 3.6.2 Molecular functions (MF)

The molecular function analysis showed that the hub genes were significantly associated with proteoglycan binding and endopeptidase activity. Proteoglycans are important components of the extracellular matrix and cell surface, and their binding may be involved in modulating cellular adhesion and migration, processes that are critical in cancer metastasis and the spread of leukemia. Endopeptidase activity is also relevant in regulating proteolytic processes that can influence tumor progression and the activation of immune cells, which is essential for cancer therapy. By modulating these activities, APS may promote more efficient immune responses and potentially reduce tumor invasiveness in AML.

#### 3.6.3 Cellular components (CC)

The enrichment analysis showed significant involvement of the hub genes in membrane rafts and vacuolar lumen, both of which are critical for immune cell function. Membrane rafts are specialized membrane microdomains that play essential roles in signal transduction and immune cell activation, while the vacuolar lumen is involved in processes such as autophagy and the degradation of cellular debris. These components are particularly relevant in the context of leukemia, as they may be involved in the activation of immune cells (such as T cells and NK cells), which are essential for recognizing and attacking leukemic cells.

#### 3.6.4 KEGG pathways

The KEGG pathway analysis highlighted several pathways that are directly involved in AML pathogenesis, including apoptosis, primary immunodeficiency, and hepatitis B. The apoptosis pathway is of particular relevance in leukemia, as the ability to promote or inhibit apoptosis is a fundamental mechanism in the treatment of cancers like AML. By modulating apoptotic pathways, APS may enhance the elimination of leukemic cells while reducing chemotherapy resistance. The primary immunodeficiency pathway also holds significance, as many childhood leukemias, including AML, are associated with immune system dysfunction. APS’s potential to influence immune signaling pathways could help reprogram the immune response, enhancing the body’s ability to fight leukemia and possibly reducing the incidence of relapse.

### 3.7 Differential expression validation and ROC curve analysis

**FIGURE 7:**
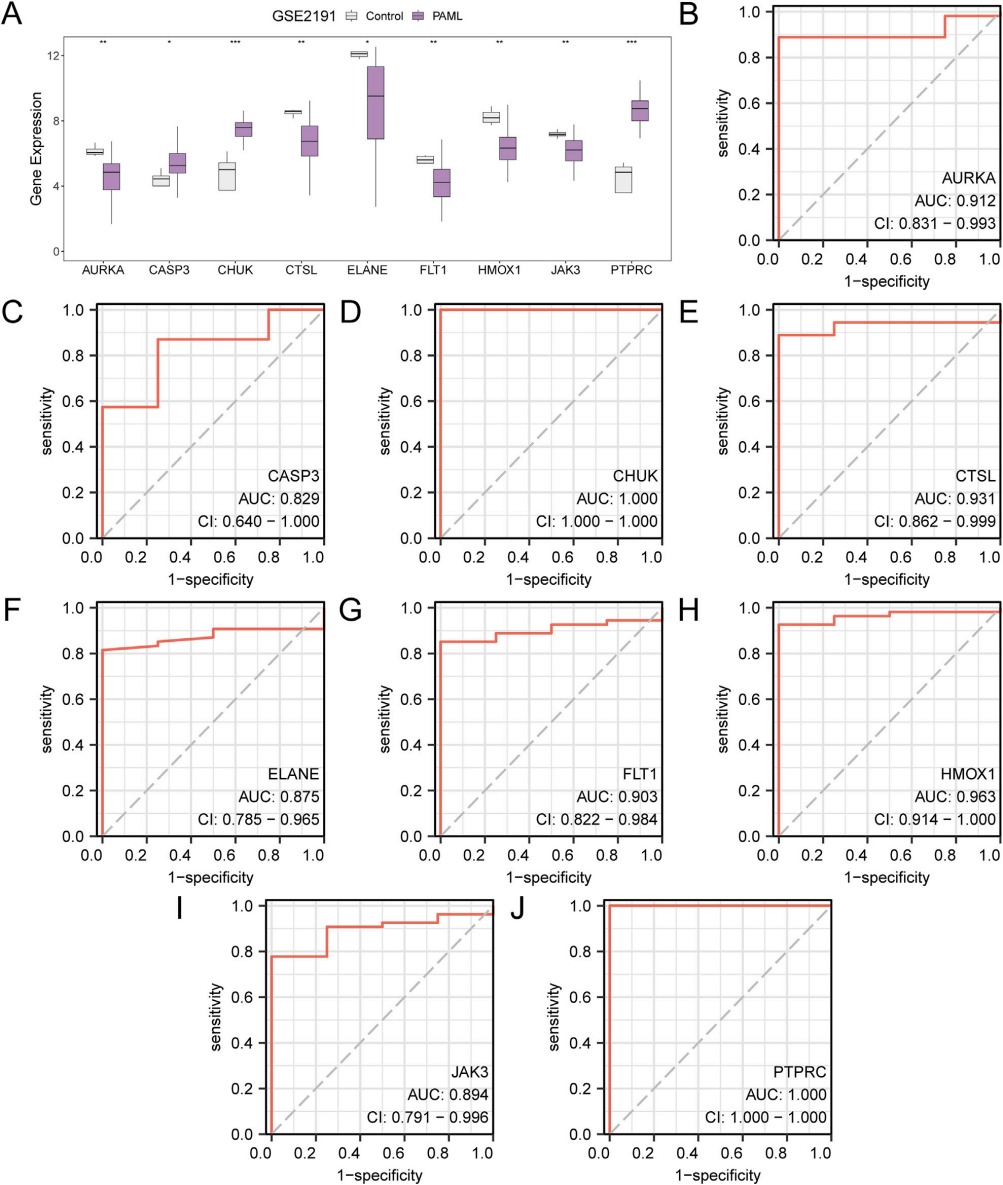
Differential Expression Validation and ROC Curve Analysis. **(A)** Group comparison diagram of Hub Genes in the PAML and the Control groups of dataset GSE2191. B-j. ROC curves of Hub Genes AURKA **(B)**, CASP3 **(C)**, CHUK **(D)**, CTSL **(E)**, ELANE **(F)**, FLT1 **(G)**, HMOX1 **(H)**, JAK3 **(I)**, PTPRC **(J)** in dataset GSE2191. * stands for p value < 0.05, indicating statistical significance; ** represents p < 0.01, highly statistically significant; *** represents p < 0.001 and highly statistically significant. When AUC > 0.5, it indicates that the expression of the molecule is a trend to promote the occurrence of the event, and the closer the AUC is to 1, the better the diagnostic effect. AUC between 0.7 and 0.9 had a certain accuracy, and AUC above 0.9 had a high accuracy. ROC, Receiver Operating Characteristic; AUC, Area Under the Curve. TPR, True Positive Rate; FPR, False Positive Rate. Gray represents the Control (Control) group and purple represents the pediatric acute myeloid leukemia (PAML) group.

The expression of hub genes in the PAML and control groups were analyzed, with all nine genes showing significant differential expression (*p* < 0.05), which are CASP3, PTPRC, ELANE, HMOX1, CHUK, FLT1, JAK3, CTSL, AURKA. ROC curve analysis using the *pROC* package revealed high classification accuracy (AUC > 0.9) for six genes (*PTPRC*, *HMOX1*, *CHUK*, *FLT1*, *CTSL*, *AURKA*), while *CASP3*, *ELANE*, and *JAK3* demonstrated moderate accuracy (0.7 < AUC < 0.9) (Figures 7A–J).

### 3.8 Hub gene correlation and functional analysis

**FIGURE 8:**
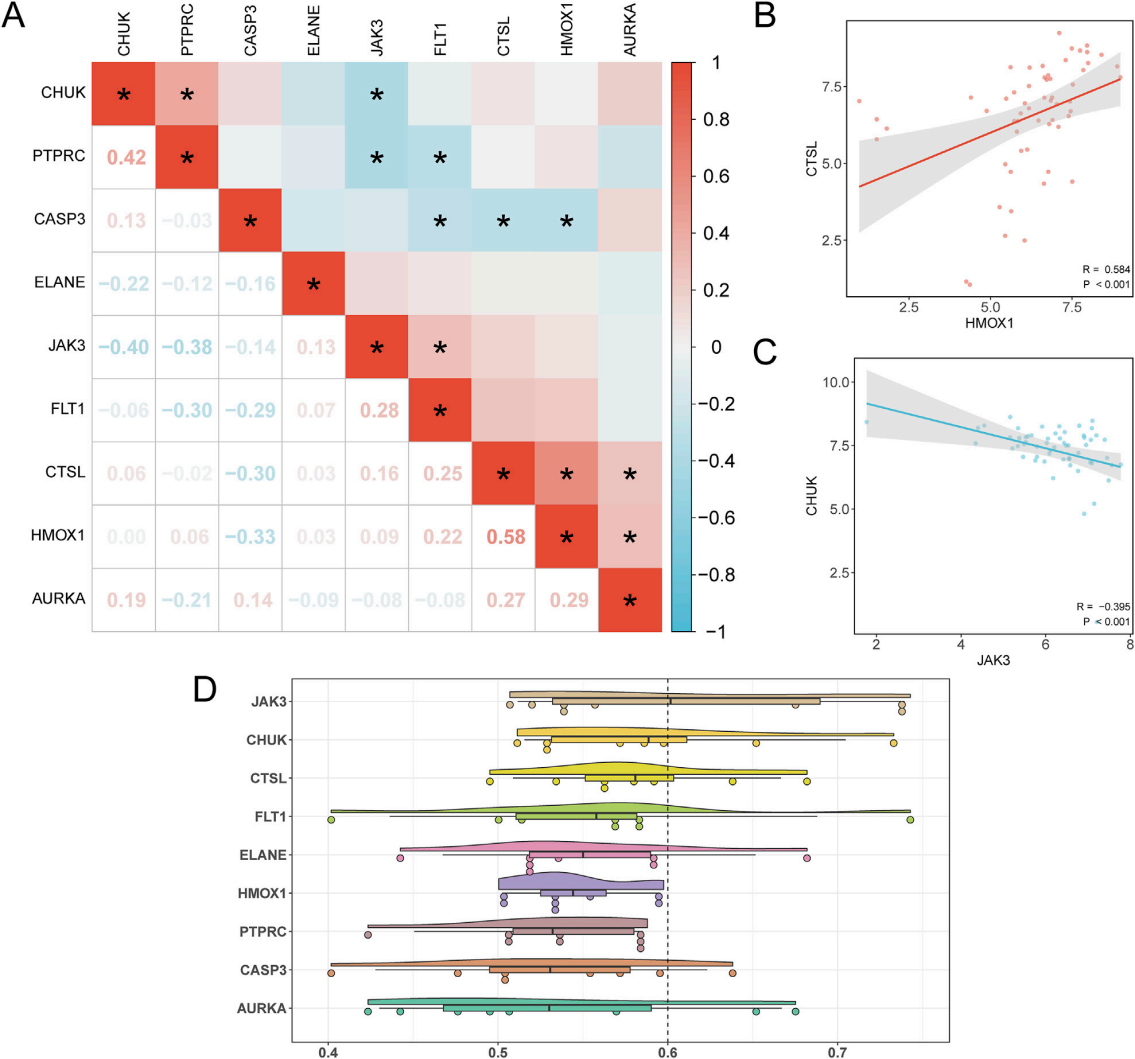
Correlation and Friends Analysis. **(A)** Correlation heatmap of Hub Genes in the PAML group and the Control group of dataset GSE2191. **(B)** Scatter plot of correlation between Hub Genes HMOX1 and CTSL. **(C)** Scatter plot of the correlation between the Hub Genes JAK3 and CHUK. **(D)** Cloud rain plot of functional similarity (Friends) analysis results of Hub Genes. **(B)** * represents p < 0.05, indicating statistical significance. The absolute value of correlation coefficient (r value) below 0.3 was weak or no correlation, 0.3 to 0.5 was weak correlation, and 0.5 to 0.8 was moderate correlation. In the group comparison diagram, purple is the PAML group, and gray is the Control group. In the correlation heat map, red is positive correlation, blue is negative correlation, and the depth of color represents the strength of correlation.

Pairwise correlations among the nine hub genes were calculated, revealing the strongest positive correlation between HMOX1 and CTSL (r = 0.584, p < 0.05), and the strongest negative correlation between JAK3 and CHUK (r = −0.395, p < 0.05) (Figure 8A). To further illustrate these findings, scatter plots were generated using the ggplot2 package to depict the strongest positively and negatively correlated gene pairs (Figures 8B, C).

In addition, functional similarity analysis was performed to evaluate the biological significance of these genes in PAML. The results, visualized in Figure 8D, highlight JAK3 as playing a critical role in the biological processes underlying PAML.

### 3.9 Immune infiltration analysis in PAML

**FIGURE 9:**
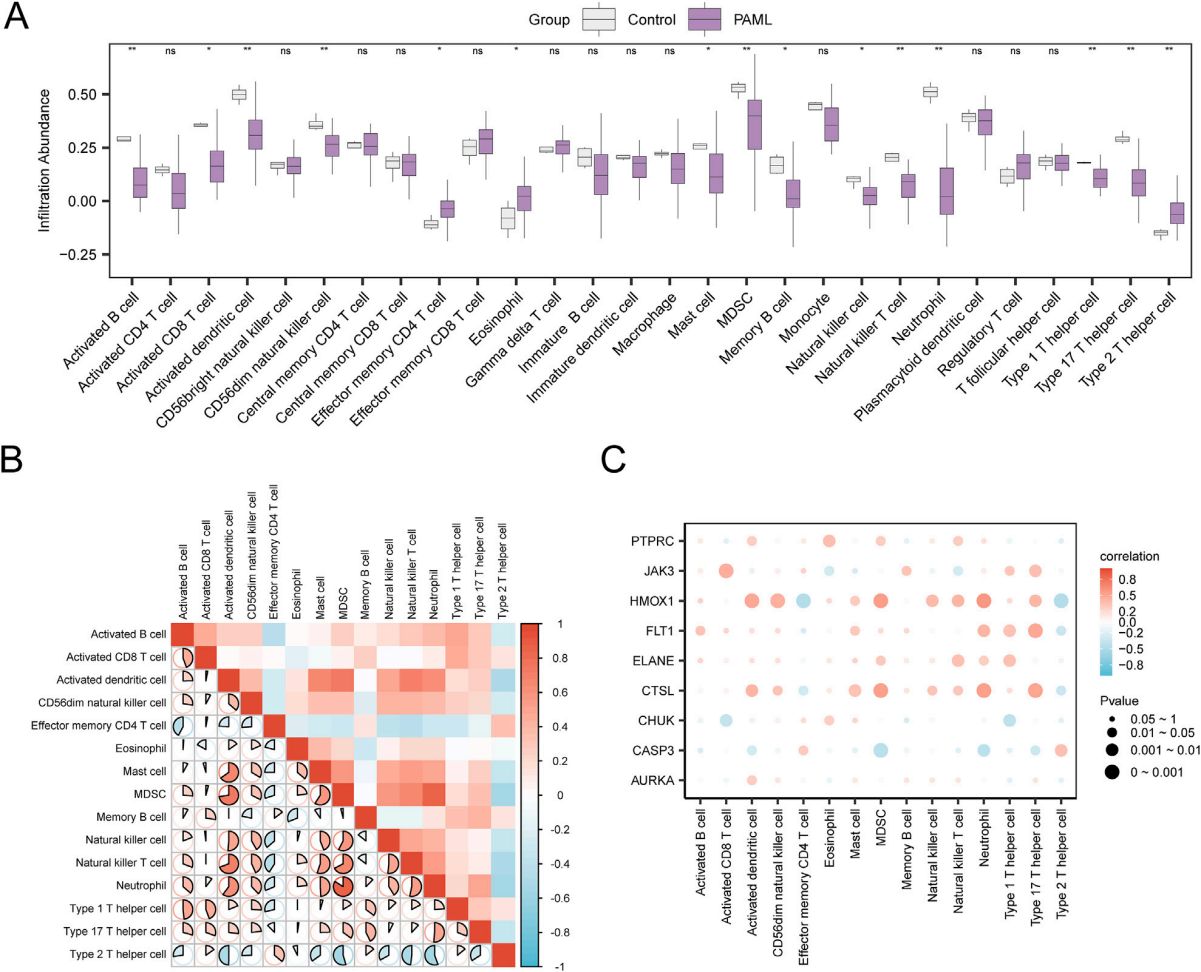
Immune Infiltration Analysis by ssGSEA Algorithm. **(A)** Grouping comparison diagram of immune cells in the Control group and the PAML group of dataset GSE2191. **(B)** Correlation heatmap of immune cell infiltration abundance in dataset GSE2191. **(C)** Bubble plot of the correlation between Hub Genes and immune cell infiltration abundance in dataset GSE2191. ssGSEA, single-sample Gene-Set Enrichment Analysis; ns stands for p ≥ 0.05, no statistical significance; * represents p < 0.05, indicating statistical significance; ** represents p < 0.01 and is highly statistically significant. The absolute value of correlation coefficient (r value) below 0.3 was weak or no correlation, 0.3 to 0.5 was weak correlation, 0.5 to 0.8 was moderate correlation, and above 0.8 was strong correlation. Control group (gray), PAML group (purple). Red shows a positive correlation, and blue shows a negative correlation. The depth of the color represents the strength of the correlation.

Using the ssGSEA algorithm, immune infiltration analysis of GSE2191 revealed 15 immune cell types with significant abundance differences (p < 0.05), including activated B cell, activated CD8^+^ T cell, activated dendritic cell, CD56 dim natural killer cell, effector memory CD4^+^ T cell, eosinophil, mast cell, myeloid-derived suppressor cell, memory B cell, natural killer cell, natural killer T cell, neutrophil, type 1 T helper cell, type 17 T helper cell, type 2 T helper cell (Figure 9A). Then, the correlation heatmap was used to show the correlation results of the abundance of 15 immune cell infiltration in the immune infiltration analysis in dataset GSE2191 (Figure 9B). The study revealed that most immune cell pairs displayed strong positive correlations. Notably, MDSCs and Neutrophils exhibited the strongest significant positive correlation (r = 0.84, p value < 0.05). Finally, Finally, the relationship between Hub Genes and immune cell infiltration abundance was illustrated using a correlation bubble plot (Figure 9C). The results indicated that many immune cells showed significant correlations with Hub Genes, with HMOX1 and Neutrophils demonstrating the strongest positive correlation (r = 0.592, p < 0.05).

## 4 Discussion

Acute myeloid leukemia (AML) is a malignant neoplasm originating from myeloid stem cell precursors, primarily affecting white blood cells, distinct from erythrocytes, platelets, B cells, and T cells. The disease arises from genetic mutations that drive abnormal clonal proliferation and tumor formation. AML accounts for approximately one-third of all leukemia diagnoses and is characterized by immune dysregulation due to underlying genetic abnormalities. Despite advancements in oncology, the treatment paradigm for AML has remained largely unchanged for nearly 50 years, with induction chemotherapy and remission therapy as the primary approaches. Our research focuses on modulating immune function as a novel therapeutic avenue to improve patient survival outcomes.

This study investigates the immunomodulatory effects of APS in pediatric AML, highlighting its potential to reshape the immune microenvironment and influence prognostic outcomes. PAML is often associated with poor survival rates and high relapse risks, necessitating the development of therapies beyond conventional chemotherapy (Hu et al., 2016; Zhang et al., 2018). APS, known for its immune-enhancing properties, emerges as a promising adjunct therapy, potentially fortifying the immune system to combat leukemic cells effectively.

APS treatment was associated with significant changes in the expression of genes involved in apoptosis and immune cell activation, notably CASP3, PTPRC and ELANE (Porter and Jänicke, 1999; Hussar, 2022; Chua and Laurent, 2006; Cohen, 1997). CASP3, a critical mediator of apoptosis, plays a role in programmed cell death, which is essential for eliminating cancerous cells. These findings align with studies indicating that APS can trigger apoptosis in various malignancies. The modulation of PTPRC (CD45) suggests enhanced leukocyte activity, which is vital for initiating and sustaining an anti-tumor immune response (Li P. et al., 2023; Salmond, 2024; Wong et al., 2013; Yang and Klein, 2022). The enrichment of these genes indicates APS’s role in reprogramming immune responses to target leukemic cells more effectively.

Moreover, the PPI network analysis revealed the central role of immune-related hub genes such as JAK3 and CHUK, both integral to cytokine signaling and immune regulation. JAK3 is particularly significant due to its association with cytokine-driven immune cell activation, suggesting that APS may enhance the proliferation and activity of immune cells targeting AML cells (Yamaoka and Kitamura, 2023; Li G. et al., 2023).

The ssGSEA analysis demonstrated that APS treatment increases the infiltration of immune cells such as activated CD8^+^ T cells, natural killer (NK) cells, and dendritic cells, which are crucial for anti-tumor immunity (Li et al., 2024). These cells contribute to direct cytotoxic activity and facilitate adaptive immune responses through antigen presentation (Smith et al., 2021). Moreover, our research revealed correlations between immune cells (e.g., CD8^+^ T cells, MDSCs, Neutrophils, and Type 17 T helper cells) and hub genes such as JAK3, CTSL, HMOX1, and FLT1. GO and KEGG enrichment analyses further supported these findings, highlighting APS’s influence on pathways involved in the negative regulation of leukocyte activation and immune-mediated apoptosis. Pathway analyses also identified APS’s role in modulating serine/threonine kinase activity and cytokine-mediated signaling, essential for coordinating complex immune responses against AML cells. This broad immune modulation aligns with traditional Chinese medicinal principles of multi-target and multi-pathway treatment strategies (Yang et al., 2024; Zhang et al., 2021). These findings support the hypothesis that APS has the potential to improve the efficacy of current AML treatments and reduce relapse rates by modulating the tumor microenvironment and immune system.

APS’s ability to enhance immune cell infiltration within the AML microenvironments offers a complementary strategy to conventional therapies, which primarily aim to eradicate leukemic cells. While conventional treatments mainly focus on eradicating leukemic cells, APS provides a complementary approach by boosting immune responses, potentially leading to improved patient outcomes. However, caution is warranted, due to potential risks such as autoimmune reactions or cytokine release syndrome arising from broad immune activation (Cosenza et al., 2021).

There are still some defects in our research. Further research is needed to optimize APS dosing to balance therapeutic efficacy with minimizing immune-related side effects. Clinical trials are essential to validate APS’s effects on pediatric AML patients and to establish its safety profile. Additionally, exploring the synergistic potential of APS in combination with other immunotherapies or targeted treatments may pave the way for personalized and effective treatment strategies.

## 5 Conclusion

In conclusion, this study provides compelling evidence that APS represents a promising adjunctive therapy for pediatric AML. By modulating immune cell infiltration and influencing key apoptotic and immune pathways, APS demonstrates the capacity to enhance anti-tumor immunity, thereby offering a promising avenue for improving patient outcomes in this challenging disease. Despite these encouraging findings, further rigorous clinical trials and in-depth mechanistic studies are imperative to fully elucidate APS’s therapeutic potential and ensure its safe integration into current treatment protocols.

### 5.1 Limitations of the study

While this study provides valuable insights into the potential molecular mechanisms of Astragalus polysaccharide (APS) in pediatric acute myeloid leukemia (PAML), several limitations must be acknowledged.

Firstly, while we analyzed the composition of APS and provided chemical formulas, these formulas represent a simplified version of the polysaccharide structure. Given that APS is a complex polysaccharide consisting of multiple monosaccharide units, the provided chemical formulas do not fully capture the intricate structure of APS. This simplification may hinder a complete understanding of the polysaccharide’s biological activity and function, and further structural elucidation is necessary in future studies to better characterize its molecular complexity.

Secondly, the sample sources used in this study, specifically the publicly available GEO dataset (GSE2191), may limit the generalizability of the findings. The dataset predominantly includes samples from a specific patient cohort and may not fully represent the broader spectrum of pediatric AML cases. Future studies should incorporate a more diverse set of samples, potentially from different populations and with varying plant sources and extraction methods, to assess whether the observed findings hold across different clinical and biological contexts.

Another limitation is the lack of in-depth exploration of the specific molecular mechanisms of APS in PAML. While we have provided a bioinformatics analysis of APS-related gene expression, the precise molecular pathways through which APS exerts its effects remain unclear. Further research is needed to specifically elucidate the mechanisms underlying the biological activities of APS, including its interactions with immune cells, signaling pathways, and other molecular macromolecules.

Additionally, although our study leveraged bioinformatics analysis of publicly available datasets, we recognize that some of these datasets lack detailed clinical information, such as treatment regimens and patient outcomes. This absence of clinical data limits the direct clinical applicability and translation of our findings. However, the primary goal of this study was to explore the molecular mechanisms of APS in pediatric AML, rather than conduct a comprehensive clinical correlation analysis. Despite these limitations, we were able to derive meaningful conclusions based on rigorous computational methods.

Finally, to strengthen the clinical relevance of our findings, future research will aim to integrate datasets with more comprehensive clinical information, allowing for better validation of our conclusions and exploring the potential clinical applications of APS in the treatment of pediatric AML.

## Data Availability

The datasets presented in this study can be found in online repositories. The names of the repository/repositories and accession number(s) can be found in the article/Supplementary Material.
