# Supplementary material for: Evaluating the role of astragalus polysaccharide in modulating immune infiltration and enhancing prognostic biomarkers in pediatric acute myeloid leukemia
Source: Front Pharmacol. 2025 Apr 7;16:1538888. doi: 10.3389/fphar.2025.1538888 (PMC12009888; doi:10.3389/fphar.2025.1538888)
Supplement: Supplementary file 1 [file Table1.docx]

Supplementary Material

## Supplementary Table

## Table S1 Astragalus Polysacharin-Related Genes（APSRGs）

|  |  | **Table S1** |  |  |  |
| --- | --- | --- | --- | --- | --- |
| PDE7A | ELANE | CASP9 | ADORA2A | TLR9 | PDE4A |
| EGFR | PLAU | CASP3 | GSK3A | CYP11B1 | PDE4C |
| PDE4B | GSTP1 | ADORA3 | PIP4K2C | CYP11B2 | GABRA3 |
| PDE4D | GSTM2 | CASP6 | FABP4 | CA9 | GABRG2 |
| PDE7B | MGLL | CASP7 | ALOX15 | MAPK1 | GABRB2 |
| PTPRC | PLA2G10 | CHUK | TTR | PRMT3 | CCNA2 |
| TGM2 | TERT | DAO | SIRT1 | PRKCG | CES1 |
| TOP2A | ABCG2 | FLT1 | MTNR1B | TRPM8 | CASP4 |
| DCTPP1 | MET | TYMP | CA2 | CCND1 | KAT2B |
| ALPL | CA12 | PTGES | GABRB3 | CDK2 | ILK |
| ERBB2 | CES2 | TAAR1 | GABRA2 | SLC6A3 | CDC7 |
| ALOX5 | MERTK | CLK1 | CA1 | NOS3 | ACHE |
| PTGS1 | DYRK1A | LRRK2 | HSD11B1 | NAAA | DYRK1B |
| PTGS2 | CTSV | KDR | GABRA1 | NR3C2 | AURKA |
| NQO2 | CTSL | HMOX1 | PGR | JAK3 | CHEK1 |
| BCHE | DYRK2 | EPAS1 | MPI | SLC9A1 | CA7 |
| F2 | MKNK1 | KLKB1 | GABRA5 | CA5A |  |
